# Communication and engagement as potentiality in everyday life between persons with young onset dementia living in a nursing home and caregivers

**DOI:** 10.1080/17482631.2022.2035305

**Published:** 2022-02-08

**Authors:** Mandana Fallahpour, Louise Nygård, Eric Asaba

**Affiliations:** a Department of Neurobiology, Care Sciences and Society (Nvs), Division of Occupational Therapy, Karolinska Institutet, Stockholm, Sweden; b Unit for Research, Education, Development, & Innovation, Stockholms Sjukhem Foundation, Stockholm, Sweden; c Department of Health Sciences, Occupational Science and Occupational Therapy Research Group, Lund University, Lund, Sweden

**Keywords:** Young-onset dementia, caregivers, communication, everyday life, nursing home

## Abstract

**Objectives:**

To explore communication and engagement in everyday situations between persons with young-onset dementia (YOD) living in a nursing home (NH) and the caregivers.

**Methods:**

The study draws on ethnographic methods aligned with participatory design. Three residents with YOD living in a NH and eight staff members were recruited. A narrative approach was used for data collection and analysis.

**Findings:**

Three narrative vignettes were developed representing everyday situations in which communication and engagement was enacted among residents and caregiver staff: (a) waiting for something to happen, (b) tensions about everyday communication, and (c) negotiating a combined living + working environment. The findings stress a paradoxical tension rooted in the NH as residence and workplace as well as place of calm and place of boredom. The everyday situations are interpreted differently from the perspective of the residents and staff.

**Conclusion:**

The identified paradox of planned and spontaneous situations influences communication and engagement in everyday life, and the potentiality for active engagement embedded in contexts of units for residents with YOD. The degree to which everyday activities and encounters are redefined and renegotiated is an important part of caregiving practices in NH settings for residents with YOD.

## Background

Dementia is one of the major causes of disability and dependency among older adults worldwide with physical, psychological, social and economic impact on caregivers, families and society (World Health Organization, 2017). Research has illustrated how persons with dementia in nursing home (NH) settings appear passive and risk a lack of engaging activities (Gustavsson et al., 2015; Holthe et al., 2007; Wood et al., 2009). Engaging in everyday activities for residents in NH settings often requires communication and cooperation among multiple stakeholders (Mondaca et al., 2018). In response to this, the role of facilitating ethical and responsive engagement in everyday activities for residents in NH settings can be supported through partnerships between residents, caregiver staff, and sometimes family members (Mondaca et al., 2018); moreover, resourcefulness can be built on in order to engage residents in the NH contexts (Mondaca et al., 2019). Furthermore, applying the concept of potentiality to communication and engagement in everyday life here shifts a certain focus away from the individual person and instead focuses on situations in which the person is engaged (Asaba & Wicks, 2010). However, prior research has shown that staffs’ perceived strain can negatively impact on the quality of caregiving services they provide, which in turn impacts on the everyday lives among persons with dementia, and how they engage in everyday life since their engagement happens in the context of communication with the caregivers (Edvardsson et al., 2008). This indicated that the developing and fostering of ongoing partnerships in dementia care constantly needs to be negotiated in each context (Edvardsson et al., 2009). Previous research has also identified a caring climate, staff education, reflective practice and staff age as predictors of job strain among caregiver staff in NH settings (Edvardsson et al., 2009).

In this study of everyday life in a NH setting, the focus is particularly on residents with Young-Onset Dementia (YOD), which has been defined as persons with dementia who are younger than 65 years (Alzheimer’s Society, 2015). Persons with YOD are estimated to constitute 5–9% of all persons with dementia (World Health Organization, 2012) YOD may present differently than late-onset dementia (LOD) in terms of non-cognitive neurological symptoms. Previous research shows that persons with YOD experienced a broad range of neurological features in association with progressive cognitive decline e.g., Chorea Parkinsonism, Seizure, motor dysfunction, sensory changes, cerebral dysfunction an visual impairment (Kelley et al., 2009). Research also shows that everyday life of persons with YOD generally differ from those of older persons with dementia, as they might have children still living at home, be physically competent, and still work (Baptista et al., 2016). Persons with YOD may therefore have different needs for support (Alzheimer’s Society, 2015) and their demands for care services may consequently be different (Millenaar et al., 2016; Williams et al., 2001). Specific problems related to active life e.g., roles and responsibilities in everyday life, and work life, social roles, family relationships, and need of stimulating activities and social contacts differ for persons with YOD as compared to those who are older than 65 years (Ducharme et al., 2014; Millenaar et al., 2016). Evidence shows that it is also challenging for person with YOD to get access to appropriate age-related services such as long-term residential care (Carter et al., 2018; Rodda & Carter, 2016). At the same time, the NH settings have difficulties in meeting the needs of persons with YOD since these settings have been designed with a focus on the needs of older residents (Carter et al., 2018). Having meaningful daytime/daily activities was identified as one of the most unmet needs reported for persons with YOD (Bakker et al., 2014). Moreover, previous research stressed the importance of providing opportunities for persons with YOD by caregivers to enhance a sense of usefulness through engagement in activities (Van Vliet et al., 2017).

The literature suggests that evidence building on experiences of younger people with dementia remains limited, and that this population is continually under-represented in the literature. Building on previous research (Edvardsson et al., 2008, 2009; Mondaca et al., 2019, 2018), actively incorporating residents and caregiver staff in joint decision-making and empowerment can improve participation and engagement in everyday life among residents with dementia. Moreover, previous research mostly focuses on services designed specifically for older adults with dementia while there is still a lack of knowledge addressing the needs of persons with YOD living at the NH setting (Millenaar et al., 2016). Furthermore, more knowledge is needed in how to actively co-create opportunities for communication and active engagement in everyday life through building common horizons of understanding within eldercare contexts. The aim of this paper is to explore communication and engagement in everyday situations between persons with YOD living in a NH and the caregivers.

## Methods

### Design

The study draws on ethnographic methods aligned with participatory design in order to discover, engage, and learn about how everyday situations work in a NH setting (Smith, 2006). The participatory design (Asaba & Suarez-Balcazar, 2018) was applied for planning and conducting the research process in the study to promote active involvement of the study participants (e.g., to guide the staff in planning, recruiting the residents, applying photography as an activity conducted by the residents and caregiver staff, and interviewing the participants). A narrative approach wa

s used for both data collection and data analysis (Burner, 1990; Polkinghorne, 1995, 2005). Because the researcher who gathered data (the first author) was not a familiar person for the participants with YOD, several sessions of ethnographic hanging out were part of the design prior to initiating interviews. Moreover, in order to capture multiple perspectives around shared situations, interviews and group sessions were conducted with persons with YOD and caregivers. This means that narratives in this study were a co-construction through understanding given situations brought up by persons with YOD. The study was approved by the regional ethics board (Dnr: 2013/1369-31/5). All participants gave their informed consent, and for persons with YOD informed consent was also obtained from a legal proxy if relevant. All aspects of the research process were carried out in accordance with ethical principles and guidelines from the national ethics authority.

### Participants and data collection

The study planned to recruit both residents and staff as participants. According to the participatory design of the study, the staff were involved in the process of recruitment and data collection. The term staff members in this study refers to caregiver staff, management staff, and health professionals in the rehabilitation team staff. When we refer to specific staff members, we qualify i.e., caregiver staff. As the first step, the study was presented by the first and last authors (MF and EA) for the management staff, and then the caregiver staff at a unit for persons with YOD at a NH in Stockholm. A purposive sample of three persons with YOD (age <65) and four caregiver staff were originally recruited at the unit for persons with YOD, who were (i) interested in participating in the study, (ii) able to take photos, and to sit and talk in the group, and (iii) to share their experiences verbally. Of the eight residents at the unit, three persons met the inclusion criteria and were recruited in the study. During data gathering, further key participants were identified and thus another five staff members and one family member were successively included in interviews. The three resident participants in the study were men aged less than 65 with a Swedish background. The successive recruitment strategy is in line with ethnographies and is relevant as an approach in identifying people who can contribute with experiences and perspectives that illustrate everyday situations in the setting. The staff members included four caregiver staff, two persons in the management staff, and two health professionals from the rehabilitation team (i.e., occupational therapist, physiotherapist). All eight staff participants were women (40–60 years old) with a Swedish background who had formal education in dementia care. The caregiver staff had extensive experiences in dementia care among both persons with LOD and YOD. An overview of the study participants is presented in Table I.

**Table I.** An overview of the study participants (n = 11)

Participants	n	Characteristics
Residents with YOD	3	Male age < 65 Swedish
Caregiver staff	4	Female Formal education within practical nursing, age between 40–60 years Swedish College educated and experienced within dementia care
Management staff	2	
Health professionals from the rehabilitation team staff (OT, PT)	2	

OT = occupational therapist; PT = physiotherapist

Data were collected through interviews (individual or group), participant-generated photos, and participant observations. An overview of the data collection sessions is presented in Table II.

**Table II.** An overview of the data collection sessions in the study

Data collection method	Description
Interview (individually or in pair)	Nine interviews
Observation	Twice a week during the day (9:00–16:00) between September and December
Photos	Thirty-one photos
Group discussion as a photovoice session	Two occasions

Initially, resident participants and the caregiver staff were asked to identify important situations of which they could take pictures and bring to the subsequent group discussion or interview session. Engaging caregiver staff and residents in a joint task of taking pictures was challenging for several reasons. For instance, even though the residents and caregiver staff engaged in a discussion about what to capture in a picture, making decisions tended to be more staff driven than a co-constructed decision process. The challenge was in no way a result of disinterest from caregiver staff, but the time and negotiation needed to accomplish co-construction in this context was difficult. Moreover, it turned out to be overly challenging for residents to remember pictures, which inhibited discussion in group.

Therefore, interviews with caregiver staff (individually or in pair) separately from individual interviews with residents coupled with participant observation were included during data collection. Moreover, to understand the context and decisions as well as activities within the context from multiple perspectives, interviews with management staff, health professionals, and a family member were also included. There was no separate interview with the family members. There was only one family member who was present during the interview of one resident. This successive recruitment of participants enriched data for analysis in accordance with narrative and ethnographic methods.

These data were collected by the first and last authors in the study. Observations were conducted through fieldwork (Smith, 2006) by the first author, twice a week, between 9:00 and 16:00 during the day. In line with the participant observations (Smith, 2006), the first author also posed questions to the participants (residents and caregiver staff) during everyday activities at the unit to better understand the unfolding situations. Each of the two sessions of group discussions to discuss the photos taken by the residents together with the caregiver staff as dyad took 60–90 minutes. Semi-structured interviews were conducted with the residents, caregiver staff, management staff and rehabilitation team staff using the interview guide (Kvale, 2007). The interviews lasted between 60–90 minutes and took place at the unit. The researchers’ focus in interviews, observations, photos, and group discussions were residents’ engagement in everyday life through their communication with the caregiver staff at the unit for persons with YOD in the NH. The situations of interest were therefore those where the residents and caregiver staff engaged in different activities together, reacted, interacted and encountered each other in different ways. The group discussions, and interviews were conducted by the first and last authors in the study.

### Study context

The study was performed in a Swedish young-onset dementia unit within a NH (8–10 residents, age < 65). Each resident had an individual room with personal furniture and material, situated along the hallway, which was adjacent to a common room with TV and sofa on one side and on the other a furnished library area with books and newspapers. Adjacent to the dining area was a kitchen, and in this common space staff and residents met for meals three times a day and coffee or snacks two times a day. The staff room was located furthest down in the long hallway with a glass wall, which was always locked from the inside. The unit had two exits, which were also locked. Occasionally there were activities organized at the unit (e.g., singing together).

### Data analysis

The Narrative analysis (Polkinghorne, 1995) in this paper draws on open interviews, participant observations, photos as well as sessions in which photos were discussed by residents and caregiver staff in a group. The photos were used as support to observations and interviews in the data analysis. All interviews were audio recorded and transcribed. In the analysis, an emphasis was on events and actions in the data gathering context and on the meaning that is enacted or narrated in relation to everyday life situations. In the data analysis, the researchers first focused on everyday situations from the perspective of nursing home residents through data generated from photos, observations, and interviews. In exploring the data, significant events situated in residents’ everyday life activities were identified. Significant events in the context of the analysis meant things that stood out for participants in the study or things that challenged prior knowledge about everyday situations in the NH. Notes were taken to record reasoning around why events were considered as significant, and to whom those events were significant. Short vignettes were written about significant events in order to contextualize the event and to understand from where it originated and where it ended, in narrative terms. Socio-cultural aspects such as values, rules, and regulations (structure and culture of the unit), and experiences were also taken into account to understand each vignette. In the analysis, the vignettes functioned as a tool in exploring emerging plots among various alternative explanations to phenomena that arose in situations. The analytical process was grounded in reflective discussions around the vignettes among the authors. The stories presented as the findings in this paper, culminate from several vignettes that have been drawn together to illustrate everyday situations in which everyday happenings unfold. Multiple perspectives are integrated in order to understand situations.

Trustworthiness and the degree to which various strategies need to be used has been debated (Morse, 2015; Spalding & Phillips, 2007), however we take the position that credibility, transferability, and dependability are broadly important (Guba & Lincoln, 1989). Specifically, these aspects were addressed by spending three months observing in the setting prior to data gathering in order to understand the setting and allow for residents to become acquainted with the first author (credibility), taking extensive fieldwork notes during data gathering that inform the narratives presented (transferability), and applying different data gathering methods (credibility and dependability), which were all seen as contributing to strengthening trustworthiness. The trustworthiness was also improved by enhancing the researchers’ understanding through dialog with participants and regular peer debriefing and discussions during the data analysis process (confirmability and credibility).

## Findings

The findings are grounded in data and presented as stories in keeping with narrative analysis. This means that empirical data and findings are integrated with concepts and interpretations. The presentation of results in a narrative way allows a contextualized representation of situations in which communication and engagement is enacted among caregiver staff and persons with YOD living in the NH. Throughout the findings, concepts such as encounters, and meetings are also used exemplify aspects of communication. Although narratives are presented thematically with one participant as the main person, the themes are presented as vignettes and were identified as relevant for all participants and contexts in this study. The three narrative vignettes were developed representing everyday situations in which communication and engagement was enacted among residents and caregiver staff, presented with the following headings in the findings: (a) waiting for something to happen, (b) tensions about everyday communication, and (c) negotiating a combined living + working environment.

### A narrative about waiting for something to happen

The first vignette reflects resident and staff participants’ perspectives in experiencing and interpreting the calmness of the unit for persons with YOD in the NH. Gabriel, a 64-year-old man who was participating in the study, was sitting in his wheelchair in the community room one day, quietly enjoying a caramel, while fishing in his pocket for another. The researcher (first author) sat down and asked about his day, to which he replied, ‛It’s pretty boring’. He continued to describe a typical day in the following way: ‛Yes, one sleeps, and then one wakes up and nothing happens. So then one eats. And then one sleeps again … it is damn boring. It’s not a little boring, it’s damn boring’. Gabriel added:

‛I wake up, get up and eat breakfast. Then I go back and shower and get myself in order. Then I go and sit here. And nothing happens here. I’m waiting for something to happen, but it never does. I wait until lunch, then we have lunch. And then after lunch, I wait again until we have dinner. Eat dinner. Nothing happens. It’s boring’. Participant 1 (Resident with YOD)

Gabriel described his everyday as neither joyful, nor challenging, but part of a chronological waiting for one thing after another, over which he experienced little control. During one interview his sister joined and helped fill in the blanks about what Gabriel used to do before moving to this unit. She described him as having an interest in social activities and in meeting other people. Another participant shared a similar sentiment: ‛Activate me’, he said. When he was asked, ‛How? What do you mean?’ he answered:

‛Yes, I do not know what it would be, but trivia is always fun. But we can’t just have it. I don’t know if one should learn something, I don’t know. Something, anyway. It just gets damn boring to just sit and watch. That I can definitely say. … And then there is the longing’. Participant 1 (Resident with YOD)

Based on multiple observations over time and interviews with residents and staff participants, through their stories, expressed a certain lack of stimulation although they were able to share ideas about what they wanted to do. In looking back at fieldnotes, the sentiment of peace and calm was also confirmed when the first and last author first visited the unit. Both sides of the corridor had been lined with closed doors leading into one-room apartments. A common community room and dining area was nested at the end of the corridor, where a few staff members were preparing lunch and one resident was watching television. The first and last author had noted in their reflective notes from the first visit, a sense of difficulty in interpreting the calmness on the unit and that there were only a few people present.

Moreover, it is relevant to also place these experiences in context of Swedish elder care where these data were generated. According to the Swedish guidelines for caring for persons with dementia disease ((The Swedish National Board of Health and Welfare, 2010) providing meaningful activity for persons with dementia as well as a feeling of safety, well-being, and familiarity is of highest priority. Despite that persons with dementia often experience difficulties in communication as a result of the disease, having access to daily encounters with others has been shown to slow cognitive decline and promote a sense of well-being during early stages of dementia (Nouchi et al., 2012). Among those with most advanced symptoms of dementia, the communicative process becomes less of an individual endeavour and more of a tightly interwoven social and interactive process to maintain personal integrity (Katz & Alegria, 2009; Ward et al., 2008). The success of social and interactive process of communication is to a high degree dependent on shared understandings of common phenomena.

Thus, when placing the observations and interview data in context, there was a paradoxical tension in the calmness on the unit. The observations and interviews with residents were indicative of a wish and need for more activation and sense of control. However, the caregiver staff communicated another perspective. Through observations on the unit and interviews with staff, we learned that for the staff it was important to ‛create a calm environment’ where demand or expectations were to be tailored to the individuals’ needs. This was planned deliberately by the unit.

‛We see quite individually so it is not possible to sort of gather a group really, but they are different individuals and then you have to try to find something based on their conditions so that the demands do not get too high, and the ambition is that they should feel as good as possible, to be able to feel that they have a home environment and that they are safe and that we actually have symptom control’. Participant 4 (Caregiver staff).

The stories among caregiver staff were often imbued with the notion that noise and lots of activities would result in feelings of agitation and anxiety among residents. Caregiver staff thus prioritized individual activities rather than the group activities, which was at times challenging for working schedule at the unit. One of the caregivers said:

‛Group activities are not possible. It would be in almost all care plans: It is not appropriate with group activity for that person who reacts like this and that. These are the ones … And then there is one who walks around and destroys everything’. Participant 6 (Caregiver staff)

Ideas and experiences about the residents that could not participate in group activities was the reason for why few organized activities took place at the unit, which in turn was part of the reason for our observation of a certain calmness there. The narrative breach in waiting for something to happen is about what has also been referred to as living in different cultures (Dwyer et al., 2009). The caregiver staff, although with all the best intentions, are acting in a culture of caring for another and doing so by focusing on a prioritized hierarchy of tasks to be completed. The residents are living in a culture of wanting to do and be in the moment, which often is not in sync with the priority of tasks among the caregiver staff. This has been described as a recurrent challenge in nursing home settings that continues to persist (Dwyer et al., 2009; Osterlind et al., 2009, 2011). That which becomes particularly potent in caregiving practices such as these is that the potentiality for engaging among persons with YOD is to a large degree impacted by another. Because the degree of agency among persons with YOD is impacted in this way, it can be relevant to explore how possibilities for doing, or not doing, can be part of the NH contexts for YOD.

On the other hand, staff were active in creating possibilities for activities off-unit such as taking a walk, baking bread, or including residents in everyday routines. For instance, on one occasion the first author observed the spontaneous emergence of shared engagement between the caregiver staff and the residents in an activity. It was about 10:00 am when Ann (caregiver staff) was watering flowers in the TV room. Steve (resident) suddenly communicated an eagerness to help. He followed behind Ann as she watered the plants. Ann handed Steve the watering can, and Steve continued watering plants. While this sequence of events was unfolding, another resident decided to help Lily (another caregiver staff) by offering to vacuum the floor. The caregiver staff regularly also involved Steve in food delivery to the unit. He was given the responsibility to go to the cafeteria before every meal (together with the caregiver staff), to pick up the food and deliver it to the unit. Albeit seemingly menial at first, this daily activity gave Steve a role to challenge his needs and everyday actions and to be part of a mutual give and take. In facilitating Steve’s engagement in daily activities on the unit, the caregiver staff supporting a sense of thriving (Baxter et al., 2019) that served as a momentous antidote to an otherwise omnipresence of boredom.

The sense of *‛waiting for something to happen’* thus has a double edge meaning on this unit for persons with YOD. On one hand an experience of few things in which to be engaged emerged as a theme characterized by boredom and lack of stimulation in the environment. On the other hand, a deliberate organizing of the environment to create calmness, and in doing so, also a space for spontaneous encounters, can be seen as a way in which caregiver staff worked to create conditions for meeting what they considered needs of the residents.

### A narrative about tensions in everyday communication

The second vignette reflects the complexity of communication and social interactions in the unit for persons with YOD, and tensions that arise spontaneously in the context of everyday activities, and the constant needs for reflection and negotiation to provide a caring and engaging environment. Steve was a 62-year-old man who had been living in the unit for eight months. He was quiet and often sought to be physically close to caregiver staff on the unit. During observations at the unit, when he was alone, he was quiet and often appeared sad. However, when he was together with caregiver staff or Gabriel, who was one of his friends at the unit, he was engaged, and the sense of sadness seemed to subside. Over time, through observations at the unit, Steve through his stories and engagement in different activities frequently expressed uncertainty about his decisions and actions. He was influenced by others, such as the staff and other residents. He was especially attached to one of the caregivers (Ann). For instance, during group sessions when photos from the week were discussed, Steve would initially answered ‛No’ if asked a question; however, with encouragement and support from Ann he managed to respond. This worked with Ann and other caregivers with whom he was close. Over the course of data gathering, it was observed that he would lean in on the side on which his caregiver was sitting and the flow in communication was also indicative of a close safe and secure relationship.

On one occasion we (first and last author) were sitting with two residents (Gabriel and Steve) and two caregivers (Anita and Karin) in a group session in which we were reviewing photos taken during the week by the residents together with one of the caregiver staff. Gabriel suddenly, and in a determined and sarcastic tone, spoke to a picture that Steve had taken with one of the caregiver staff, Anita. Gabriel said, ‛His home is so damn tidy’ and Steve who was trying to make a joke said, ‛Yes, right. Yes, he does’. Gabriel added, ‛Because you can see that here in the photo’. Even if Steve went with the flow, he looked confused and upset. He was trying to figure out what he had done ‛wrong’, or at least what he had done that amounted to the sarcasm from Gabriel. After a few seconds Steve, when looking at the same picture, whispered quietly, ‛Why did I do that?’ He looked confused and could not remember anything about taking the picture. Anita who was the one accompanying him in taking the picture said, ‛What?’ and he whispered again quietly, ‛Why did I do that?’ Anita who could not understand his question asked again, ‛Done what, photo?’ and he answered ‛Yes’. Anita then understood his question and responded, ‛I took it because it showed your interest in film. You have a great interest for film’. Even if Anita explained the picture and that occasion, Steve repeated the same question. Steve was still trying to understand why Gabriel was being so sarcastic and seemingly critical of Steve’s room. He asked, ‛Yes, but what have I done?’ Anita tried to make it clear for him that, ‛You haven’t done anything’ and then he said ‛No’, while still confused and upset, but calmer. Anita now understood and added, ‛No, don’t listen to Gabriel, he says so many strange things. Sorry’ and then she tried to support Steve by looking at him calmly and taking some moments so that he could understand the situation.

In our effort to understand how the organization worked with shaping a culture around communication, managerial staff were also consulted. One member of the management staff explained:

‛We hadn’t initially thought of having a nurse who took supervisor responsibility in the unit for younger dementia, but then there were constellations in the group, so they needed a supervisor so that they could still agree, and then recruited a Silvia sister from outside who is not in the group, who has worked a lot with younger [persons with] dementia, but also worked a lot with supervision and reflection, because there must be an openness, you have to dare to say that ‛This does not work’ or ‛When you do like this or that, it [the results] will be like this, [so,] try this’; or dare to ask how you should do to succeed, because yes, it must be prestigious and high standards; and then we have group meetings, to make sure how it works, of course with temporary staff and since it is 24 hours care, it is important that the individuals fit, having the right calmness and dare to create a relationship with those who have difficulty in communication; so we work with it a lot’. Participant 8 (Management staff).

Social contacts and interactions are dependent on cultural codes. Although Gabriel and Steve spent a lot of time together during the day talking, laughing, and making fun of other people, this socially secure relationship was challenged when the ‛making fun of others’ was turned towards Steve. Through unsupported and complicated situations where Steve could not read the cultural codes, he became confused and insecure. In this case however, the caregiver staff member understood and addressed this insecurity. The handling of the delicate situation was supported by what seemed to be a deliberate effort and perhaps grounded in previous staff training in communication. How caregiving practices and the creating of engaging activity environments are co-constructed arguably depends on a capacity for reflection in-the-moment rather than preunderstanding and reifications of illness stereotypes (Katz & Alegria, 2009; Yang et al., 2007). The complexity of communication and social interactions on a unit for younger adults with dementia, where tensions arise spontaneously and throughout a continuous flow of everyday activities, needs to constantly be negotiated and renegotiated.

One way that a caregiver staff member worked to meet this need was to capture spontaneous moments of subtle communication. She illustrates a sense of tacit knowledge about how to facilitate encounters, and in this case referring to photos that were taken by the resident:

‛I can never ask for anything. Then if it happens, by chance, that, say Steve and I just start talking about what is in the picture, that’s another thing. But then it comes from him. Not from me, so … it isn’t possible to book a time for such, for example. It is completely impossible. Well, that’s … That’s when he feels that, that, that he can be himself with me. Like I constantly make it easier for him so that he can be himself and not play a game like and hide his illness for example, and so on. But when he notices that I see and attend to him, so, so, then he starts to express himself in his way. So, that, that, that, it happens all the time’. Participant 5 (Caregiver staff)

The degree to which everyday activities and encounters concurrently are renegotiated is an important part of caregiving practices in NH contexts for persons with YOD. These types of spontaneous encounters that led to doing something together on the unit, which was taken up by caregiver staff as enriching and valuable, enable residents’ communication in the context of engaging in everyday life. In a context of *‛everyday life’* on the unit, crucial identity work was unfolding as part of an ongoing process of creating, recreating, and upholding identities. Findings from myriad earlier research supports that the dignity of identity work is performed in the interaction between the individual and their physical, social, and cultural environment (Öhlander, 2009).

The findings support the claim that ‛humans are occupational beings with a need to use time in a purposeful way. The need is innate and related to health and survival because it enables individuals to utilize their biological capacities and potential and thereby flourish’ (p. 23; Wilcock, 1993, 1998). The concept of doing provides mechanism for social interaction and social development (Wilcock, 1999). The residents in the study experienced their everyday life low stimulating, giving them less opportunities for doing and not challenging and boring. The concept of being captures notions as nature and essence, about being true to ourselves, to our individual capacities and in all that we do (Wilcock, 1999). The concept of becoming adds to the notion of future, which is dependent on individuals’ doing and being in their present and their history in terms of cultural development (Wilcock, 1999).

### A narrative about negotiating a combined living + working environment

The third vignette addresses the importance of strategies to create an environment in which residents felt at home in their spaces while caregiver staff and management sharing this space had a reasonable work environment. During an occasion of observation when the researcher (first author) was at the unit, in the morning, a few of the residents and caregiver staffs were in the living room. The caregiver staff was taking care of flowers, cleaning up and redecorating the room. The residents were watching TV or sitting on the sofa. Steve and another male resident Bob were helping the caregiver staff. They were talking and working together, communicated kindly and were engaged in the activities they were doing. All of sudden, Bob left the living/ TV room to his room and rushed back in a couple of minutes with a photo of him and his wife in a frame showing it to one the caregiver staff asking: ‛When does Melissa come to pick me up, home?’ The caregiver staff responded, ‛She comes’. He repeated the same question two more times and got the same answer again. Then he said, ‛Tomorrow?’, and the caregiver staff responded, ‛Yes’. He continued, ‛When does Melissa come?’, and she answered again ‛Tomorrow’. His deep desire for getting back home presented itself even in another occasion when the researcher (first author) visited him in his room to discuss the photos he took together with a caregiver staff. He took photo of a car on the street which was similar their (his and his wife) car. The same car when his wife usually drove to visit him and took him home with her. During researcher’s visit he didn’t say anything more except he asked the caregiver staff about when his wife, Melissa, would come to take him home and each time the caregiver staff responded that she would come ‛Tomorrow’.

For the caregiver staff and management staff, it was important to create a home-like environment for the residents. Younger residents with dementia were often physically capable of being active, express a need live in a more stimulating environment to provide opportunities to be engaged in community-based activities, and were accustomed to other types of home environments than an older generation, providing a broader array of physical daily activities. This created a challenge because on one hand staff wanted to support residents as if they were in a home-like environment, and on the other hand, there was not enough staff to customize caregiving one on one.

‛So, what does dementia care need in the future? Yes, there is so much needed I think, environment if you think of it as a residence, and what is considered as cosy and home-like [hemtrevligt in Swedish], seems different for everyone; But if one has looked at the unit for younger [persons with] dementia, one can see that it is a boring corridor and that, it is very cold [socially]; and where is [a] home-like [environment] for those who are younger and how do they recognize that so that it really becomes home-home’. Participant 10 (Rehabilitation team staff)

Caregiver staff and management staff, alike, stressed the importance of needing to find strategies to make the environment, including apartments on the unit as well as the common areas as home-like as possible, even though the same environment was for staff a working environment.

‛I see it as if we are visiting their home, then I subconsciously know that it is a workplace too, for employees and that we have to think about so many things. But one can get this thought that we actually visit someone [residents] in their home and that it is their kitchen and living room. This shared common space, and working in that [the same environment], makes us think that it is not ‛us and them’, since I saw that obviously one made a difference, at least compared to my previous place, because one could sit and eat breakfast together with the residents there and things like that, we made no changes; We even invited relatives if we had Friday night’s events [Fredagsmys in Swedish], we did it together, and that is a good thing to work with’. Participant 9 (Management staff)

For the staff it was important to integrate different types of activities in order to stimulate and enable communication among residents and staffs. Caregiver staff felt that some activities were more effective such as dance and music, gardening, and activities that were physical, which match a better physical capacity among persons with YOD compared to persons with LOD.

‛So it is important to use those pieces, and one can then measure that the medicines can be reduced based on those pieces so that it nevertheless becomes [effective], since the medicine affects, whether the person is younger or older. Many pieces, and one can use those as tools instead, i.e., weighted blankets, tactile stimulation, music … So, the pieces I think are important, then it is important to find meaningful activities. It is at the individual level when it comes to the younger persons, but interestingly I can see that ….I have creative colleagues who nevertheless dare to try and open, and I try to stimulate too, to try to strengthen them since it has positive effects; And they are after all using the garden and planting and, depending on who these individuals are, one can be a part of this activity [gardening]’. Participant 11 (Rehabilitation team staff)

Living together in a social environment such as a unit for persons with YOD requires an advanced level of skills in organizing and facilitating a physical and social environment as well as the dynamic interactions that unfold. This living context includes people that are living as residents and others who are coming and going as workers. In this way the work of caregiving and creating of engaging activity environments depends on a capacity for reflection rather than deconstructive reifications of illness stereotypes (Katz & Alegria, 2009; Yang et al., 2007). Moreover, previous research has illustrated the importance of home as a safe familiar place for older adults where they perceive the sense of belonging and connectedness to their past and occupational identity in everyday life which is important and related to their health and well-being (Wiles et al., 2012). Previous research has also showed that different aspects of place contributes to enabling activities and collaboration between persons with dementia and staff and influencing their ability to achieve an active or passive role in everyday life (Myren et al., 2017). Engaging in meaningful social interactions plays a crucial role in everyday life among people with YOD. However, literature (Campo & Chaudhury, 2012) suggests that it is a great challenge to support social interactions and that social interactions generally decrease among persons with dementia who move into NH settings. These results stress the importance and need of adapting the social and physical environments within NH settings, to facilitate social interactions among residents and providing opportunities for their active engagement in everyday life.

## Discussion

The aim of the study was to explore communication and engagement in everyday situations between persons with YOD living in a NH and the caregivers. Previous literature focusing on older adults with dementia demonstrated lack of a stimulating everyday life in the context of NH (Gustavsson et al., 2015; Holthe et al., 2007; Wood et al., 2009). The findings of this study, using another theoretical lens with focus on persons with YOD including relevant stakeholders around them, stresses a duality and paradoxical tension experienced in the everyday situations voiced by residents and staff. The findings stress a paradoxical tension rooted in the NH as place of calm and place of boredom as identified in the first vignette as well as residence and workplace as identified in third vignette. The identified paradox of these everyday situations influences their communication and engagement in everyday life, which is grounded in individuals’ (both residents and staff) roles as a person and the context of nursing home which is not static. Consequently, paradoxical situations (vignettes 1 and 3) and tensions in communications (vignette 2) can restrict residents’ engagement in everyday life. The findings also emphasize how the work of caring and creating engaging activity environments depends on a capacity for reflection rather than deconstructive reifications of illness stereotypes (Katz & Alegria, 2009; Yang et al., 2007).

The sense of ‛*waiting for something to happen’* in the first vignette, has a double edge meaning of boredom versus calmness for the residents and staff respectively. The deliberated creation of a calm environment might make it possible for the staff to (i) create conditions for meeting what they consider as needs of residents, (ii) prevent agitation and emotional anxiety among the residents by less noise and activities, (iii) avoid the challenges and have control over their work to tailor the demands and expectations and (iv) keep the routine and culture in the NH. However, the experience of few things to be engaged in, emerged as the first vignette categorized by boredom and lack of stimulation among the residents restricts the provision of opportunities for active engagement in everyday activities and encounters.

Experiencing a passive everyday life and losing a sense of control over everyday life can be harmful to residents’ health and wellbeing (Nyqvist et al., 2013; Puvill et al., 2016). Previous research has stressed the importance of autonomy and self-determination in the experience of active participation in everyday life and engagement (Fallahpour et al., 2013). Moreover, it can be important to see these views in light of how dyads of older adult-caregiver can create situations in which everyday life is imbued with a sense of influence (Asaba et al., 2021). Previous research has also emphasized the importance of having balance in different everyday activities and the engaging activities in everyday life (Jonsson, 2011). The findings can be understood in light of the eight-channel flow model (Massimini et al., 1987), in which boredom is characterized by a medium level skill set and a low degree of challenge. From Gabriel’s perspective, passive low challenging everyday physical activities with no expectations can have resulted in a sense of boredom because he still had energy to be more physically active and a wish to do more. The imbalance between his inner motivation and need to be engaged and the external demands and expectations created a strong sense of frustration that imbued much of his narrative. Moreover the gap between what he wanted to do and what he was actually doing seemed to increase with time, an occupational gap (Eriksson et al., 2006), can also be associated with a risk for lower level of life satisfaction (Eriksson et al., 2009; Fallahpour et al., 2011)

The paradoxical meaning of place, or acting in different cultures, also deserves further exploration in that a residence as expected by the residents to be ‛home’ and a ‛workplace’ as expected by the staff inevitably creates different needs, expectations and challenges in everyday interactions. This living and working context including both residents and the staff requires facilitating a flexible physical and social environment which enable social interactions and provide a caring engaging atmosphere. The findings highlight the necessity of modifying the environment in order to facilitate a home-friendly atmosphere where the residents feel safe and where staff feel comfortable in empowering residents. Previous research has stressed the importance of home as a safe familiar place for older adults where they perceive a sense of belonging and connectedness to their past and occupational identity, and a sense of independence and autonomy in everyday life (Dahlin-Ivanoff et al., 2007; Haak et al., 2007; Wiles et al., 2012).There is however still lack of knowledge regarding how to provide such a home-friendly environment in the NH settings which necessitates future research. Although empirical literature (Campo & Chaudhury, 2012) has stressed the importance of engaging in meaningful social interactions in everyday life among persons with YOD, it is still a great challenge how to provide an engaging environment to support social interactions among persons YOD living in NH settings.

## Methodological Considerations

The study applied an ethnographic and participatory design to collect data which is the strength of the study to encourage active participation among the participants in the study. However, one of the challenges was engage residents and caregivers sufficiently in the NH setting to achieve rich data, especially among the residents, which led to an adjustment in the data collection. The original plan was to collect the data through photovoice sessions where both the residents and caregiver staff take part in the discussion. However, the photovoice session were limited to two occasions, the data were therefore generated through interviews, photos, and observations. Applying a combination of different data collection methods in this study, however, provided the opportunity for a co-construction of narratives to understand everyday life situations in which communication and engagement was enacted among caregiver staff and persons with YOD living in the NH. The presentation of the participants in this study has been limited to a group description and not the demographic characteristics of each person. This deliberate decision was made by the research group to ensure that anonymity and confidentiality of the data has been fulfilled.

## Conclusion

This study has focused on everyday situations, and reasoning around communication and engagement as potentiality in everyday life among persons with YOD living in nursing homes and the caregiver staff. The findings of this study focus on persons with YOD, the caregiver staff, the NH context, other staff members such as management staff and rehabilitation staff, who collectively stress a paradoxical tension rooted in the NH as residence and workplace as well as place of calm and place of boredom. The everyday situations are interpreted differently from the perspective of the residents, family caregivers, and staff. The identified paradox of planned and spontaneous situations influences communication and engagement in everyday life, and the potentiality for active engagement embedded in contexts of units for residents with YOD. The degree to which everyday activities and encounters are redefined and renegotiated is an important part of caregiving practices in NH settings for residents with YOD.
